# A degradable PEGDA-dopamine hydrogel with ROS scavenging capacity supports flexible design for nerve repair

**DOI:** 10.1016/j.mtbio.2026.103203

**Published:** 2026-05-05

**Authors:** Lin Huang, Ting-Yu Lu, Emma Berman, Alexander Park, Katarina Ercegovac, Jacob Schimelman, Shaochen Chen

**Affiliations:** a Department of Chemical and Nano Engineering, University of California San Diego, La Jolla, USA; b Department of Bioengineering, University of California, Berkeley, Berkeley, USA; c Department of Bioengineering, University of California San Diego, La Jolla, USA

**Keywords:** 3D bioprinting, Peripheral nerve repair, Biomaterial, Biodegradation

## Abstract

Peripheral nerve injury remains a significant clinical challenge, with current therapeutic material limited by inadequate degradation control, insufficient oxidative stress management, and poor adaptability to patient-specific contexts. We developed a degradable poly (ethylene glycol) diacrylate-dopamine-acrylamide hydrogel platform that addresses these limitations, enabling tunable bulk degradation with concomitant dopamine release. By systematically varying the ratio of degradable crosslinker poly (ethylene glycol) diacrylate-dopamine, we generated composition-defined degradation profiles spanning 2 months with corresponding dopamine release patterns. The hydrogels exhibited mechanical properties comparable to native peripheral nerves while maintaining exceptional flexibility through multiple bending and torsional cycles. In vitro validation demonstrated that dopamine-releasing hydrogels effectively scavenged intracellular reactive oxygen species in both human Schwann cells and endothelial cells under oxidative challenge, while modulating Schwann cell gene expression in a pattern consistent with a transition from repair toward a pro-remyelination transcriptional profile, and shifting endothelial gene expression toward a pro-angiogenic transcriptional pattern. Using digital light processing bioprinting we fabricated customizable nerve wraps, tubular structures, and microarchitectures with internal channels that directed cell alignment, while controlled FITC-dextran release validated localized delivery capabilities. These findings establish a multifunctional hydrogel platform combining programmable degradation, antioxidant functionality, and cellular microenvironment control for peripheral nerve repair applications.

## Graphical abstract

**Figure:**
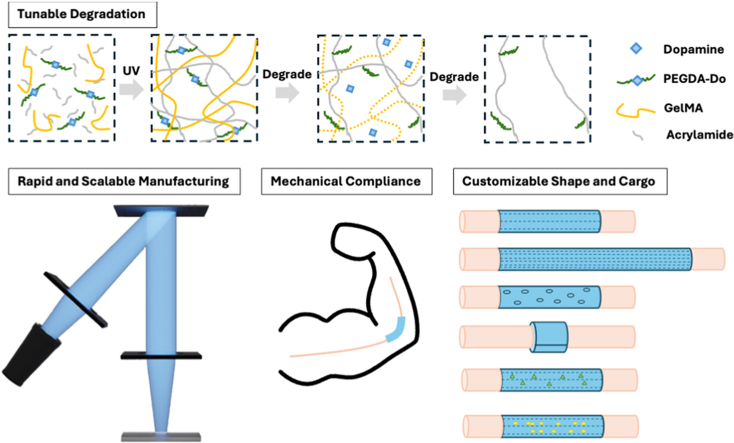

## 1 Introduction

Peripheral nerve injury (PNI) imposes a substantial clinical and socioeconomic burden and is most commonly associated with traumatic mechanisms while also intersecting with metabolic diseases such as diabetes [1]. PNI incidence is reported at ∼40 per million (upper extremity, U.S.) and 10–16 per 100,000 overall, with lasting economic burden beyond the acute phase [[2], [3], [4]]. Beyond incidence, PNI sequelae, such as motor deficits, sensory loss, and neuropathic pain, translate into long-term disability and recurrent healthcare utilization, as many as 87% of patients do not achieve full functional recovery after severe injuries such as neurotmesis [5,6].

Clinical management of PNI falls into two use cases. First, segmental loss, where tension-free coaptation is not possible, still relies on autologous nerve grafting as the reference standard, despite donor-site morbidity, limited supply, and fascicular mismatch [7]. Commercial nerve guidance conduits provide off-the-shelf alternatives, yet their consistent performance remains largely confined to short, noncritical gaps, with multiple clinical reviews noting diminished reliability beyond 1 cm in humans [8]. Second, neuropathy- or host-compromised injuries, such as those in diabetic or aging populations, present a chronically hostile milieu, including microvascular dysfunction, oxidative stress, and impaired Schwann cell (SC) support for remyelination, where even anatomically straightforward lesions recover poorly without interventions that stabilize the local niche [[9], [10], [11], [12], [13]]. These two contexts underscore that anatomical span is necessary but insufficient, repair success hinges on rebuilding and maintaining a permissive microenvironment before regenerating axons arrive.

Engineered hydrogels have rapidly expanded the therapeutic toolkit for PNI, serving as extracellular matrix (ECM)-mimetic scaffolds and local delivery vehicles that support SC function, neurite extension, and controlled biofactor release. Recent platforms span conductive networks, such as polypyrrole, reduced graphene oxide or poly (3,4-ethylenedioxythiophene)-based material, that translate electrical cues into pro-regenerative SC responses and enhanced axonal growth; peptide or self-assembling matrices, such as YIGSR, that recapitulate adhesive and guidance motifs; and composite or nanoparticle-integrated gels that sustain neurotrophin availability (nerve growth factor (NGF), brain-derived neurotrophic factor (BDNF), vascular endothelial growth factor (VEGF)) or stabilize exosomes at the lesion site for paracrine support [6,14,15]. Collectively, these systems improve SC adhesion and migration, promote myelination markers, and enable spatiotemporally tuned cargo release, yet translation remains limited and outcomes in severe defects or complex biology are inconsistent. First, many materials prioritize axon guidance or late-stage cues while under-addressing the post-injury niche, such as directing SC plasticity and establishing a permissive, angiogenic microenvironment before robust axonal ingress. Emerging work shows SC fate is strongly regulated by substrate mechanics or topography and that hydrogel-mediated trophic delivery is promising, but sustained, phase-appropriate microenvironment control is not yet routine [6,16]. Second, degradability remains difficult to program with clinical precision, as many polymer degradation processes exhibit unstable kinetics, acidic byproducts that can perturb tissue pH and inflammation, or release profiles that decouple from lesion biology, highlighting tunable biodegradation kinetics and stable, controllable release as an unmet criterion for next-generation material design for nerve repair [6,16,17]. Third, most platforms are not designed for patient-specific contexts, such as diabetic peripheral neuropathy with impaired remyelination and vascular tone [[16], [17], [18]]. Fourth, flexibility and mechanical compliance remain limiting for many reported materials. An ideal construct must provide enough strength and flexibility without pressurizing the nerve and preserve physiological movements under bending and twisting. These gaps motivate a materials strategy that programs SC and endothelial behavior, offers independently tunable degradation, is patient-contextualizable, and remains highly flexible to withstand the in vivo bending/torsion environment.

Here, inspired by Liang's work, in which a photocrosslinkerable and degradable polymer PEGDA-Dopamine (PEGDA-Do) conjugate was developed, we propose a poly (ethylene glycol) diacrylate-dopamine-acrylamide (PDAM) hydrogel in which amine-acrylamide linkages undergo reversible aza-Michael exchange, enabling degradation with concomitant dopamine release as the scaffold clears [19]. By tuning the bioink composition, specifically the PEGDA-Do ratio, we aim to generate composition-defined degradation and dopamine-release profiles that have the potential to match distinct PNI contexts. We hypothesize that dopamine liberated during degradation will buffer oxidative stress, promote endothelial sprouting, remodeling, and stabilization, and prime SC toward a repair-to-remyelination trajectory, thereby maintaining a permissive pre-axonal niche. To test this, we quantify degradation by gravimetric mass loss and track dopamine release by UV-Vis absorbance; in parallel, we investigate the mechanical properties of this hydrogel platform, assess cytocompatibility with human Schwann cells (hSCs) and human umbilical vein endothelial cells (HUVECs), evaluate expression of regeneration-relevant genes, and demonstrate geometric customizability and feature fidelity via digital light processing (DLP)-printed wraps, conduits, and microstructured architectures.

## 2 Results

### 2.1 Degradation behavior and mechanical characterization of PDAM hydrogels

To develop a hydrogel platform with tunable degradation profile, we first investigated how different bioink components, specifically the ratio between PEGDA-Do and PEGDA, affect the degradation rate. The acrylamide concentration (13 wt%) was selected following the design rationale of Yang et al. who demonstrated that PEGDA-acrylamide hydrogels with moderate monomer loading achieve rapid gelation, high print fidelity, and elastic moduli (tens to hundreds kPa) suitable for soft-tissue bioprinting [20]. This composition provides sufficient permanent-network density to stabilize the PEGDA-Do crosslinker against rapid dissolution while maintaining the flexibility and compliance required for peripheral nerve repair.

**Fig. 1:**
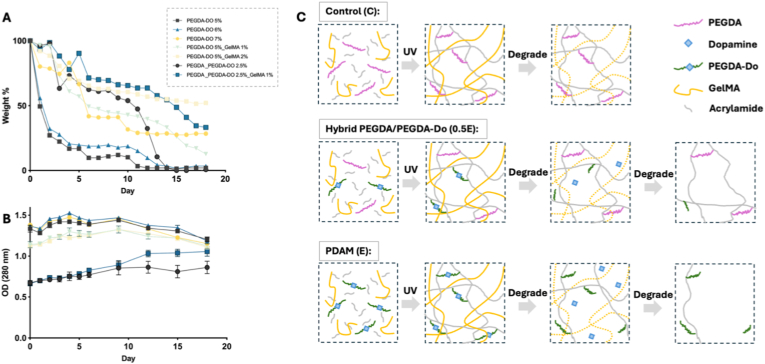
(A) Degradation profiles of hydrogels with varying compositions over 18 days (n = 3). (B) Corresponding absorbance at 280 nm indicates dopamine release during degradation, where the release rate correlates with network disassembly. (C) Schematic representation of representative hydrogel formulations: PEGDA-based control (Group C) showing slow degradation and no dopamine release; PEGDA/PEGDA-Do hybrid (Group 0.5 E) exhibiting intermediate degradation and dopamine release; and PDAM hydrogel (Group E) showing the fastest degradation and highest dopamine release. Sample sizes: n = 4.

As shown in Fig. 1A, among formulations with different PEGDA-Do composition, the 5% group exhibited the most rapid weight loss, whereas the 7% formulation degraded more slowly, suggesting that higher local crosslink density can temporarily slow down bulk dissolution despite the presence of degradable linkages. Substituting half the amount of PEGDA-Do with PEGDA prolonged the degradation duration and reduced the amount of dopamine release. To impart baseline bio-adhesiveness, we added small amounts of gelatin methacrylate (GelMA), which also modestly stabilized degradation, so GelMA was fixed at 1 wt% in all subsequent PDAM formulations while keeping the PEGDA-Do backbone as the main variable. Correspondingly, the optical absorbance at 280 nm (Fig. 1B) increased over time in PEGDA-Do-containing groups, consistent with the release of aromatic degradation products, predominantly dopamine, as the network disassembled [19]. Orthogonal quantification methods (e.g., HPLC or mass spectrometry) would be needed to determine absolute dopamine concentrations and are planned for future studies. On this basis, three representative formulations were selected for subsequent studies (Fig. 1C): the PEGDA-based control (Group C), showing the slowest degradation and no dopamine release; the hybrid PEGDA/PEGDA-Do system (Group 0.5 E), exhibiting moderate degradation and intermediate dopamine release; and the PDAM network (Group E), which displayed the most pronounced degradation and highest dopamine release overall.

**Fig. 2:**
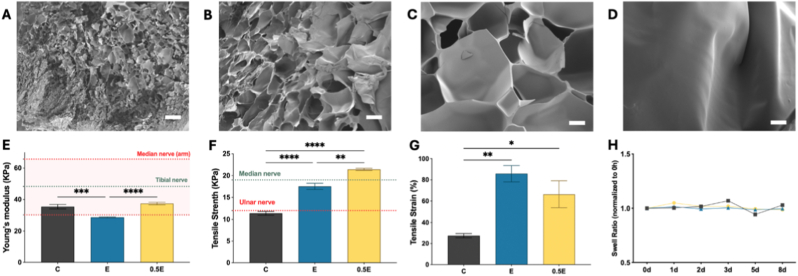
(A–C) Representative SEM images of Group C, Group E, and Group 0.5 E hydrogels, respectively, illustrating a progressive increase in pore size across formulations. Images were captured at magnifications of 100 × , 500 × , and 100 × with corresponding scale bars of 100 μm, 20 μm, and 100 μm for panels A, B, and C, respectively. (D) SEM image of a 5% PEGDA-Do hydrogel without acrylamide or GelMA. Scale bars: 100 μm. (E-G) Mechanical characterization of hydrogels showing Young's modulus, tensile strength, and ultimate strain (n = 3), compared against reported mechanical ranges of peripheral nerves [[21], [22], [23]]. (H) Swelling ratios during 8 days of PBS immersion, normalized to day 0 (mean ± SEM, ∗P < 0.05, ∗∗P < 0.01, ∗∗∗P < 0.001, ∗∗∗∗P < 0.0001).

SEM revealed distinct morphology among Group C, Group 0.5 E, and Group E hydrogels. Group C exhibited a compact and relatively dense morphology (Fig. 2A), whereas Group 0.5 E showed a more open and interconnected pore network (Fig. 2B). Group E displayed the largest pores, reflecting the longer polymer chain length between crosslinks due to the higher molecular weight of PEGDA-Do compared to Group C and Group 0.5 E (Fig. 2C). To visualize the intrinsic morphology of PEGDA-Do without supporting monomers acrylamide, a separate sample composed of 5% PEGDA-Do only (no acrylamide or GelMA) was examined (Fig. 2D). These morphological differences suggest that increasing the proportion of PEGDA-Do leads to a more porous architecture favorable for nutrient diffusion and mass transport.

Mechanical testing confirmed that all hydrogels were soft yet elastically resilient (Fig. 2E–G), with Young's modulus at around 35 kPa, comparable to human tibial and median nerves (30-65 kPa) (Fig. 2E), and tensile strengths between 10 and 20 kPa, consistent with ulnar and tibial nerve values (12-20 kPa) (Fig. 2F) [[21], [22], [23]]. Ultimate strain reached up to ∼80%, indicating that the materials can sustain substantial deformation without rupture (Fig. 2G). Swelling ratios remained stable over 8 days of PBS immersion, confirming structural integrity and hydration stability (Fig. 2H).

Since PDAM hydrogels release dopamine during degradation, and dopamine has been widely reported as a reactive oxygen species scavenging agent, we next evaluated the antioxidant capacity of the hydrogel degradation products [[24], [25], [26]]. Hydrogel-incubated medium was collected from Group C, Group 0.5 E, and Group E hydrogel slabs after 24 h of immersion, and used to culture HUVECs on tissue culture plates prior to oxidative challenge with 200 μM H_2_O_2_. As shown in Fig. S3, HUVECs cultured with Group E hydrogel-incubated medium exhibited markedly reduced DCFDA fluorescence compared with Group C, while Group 0.5 E produced an intermediate response, demonstrating that the ROS scavenging capacity scales with PEGDA-Do content in the hydrogel formulation.

### 2.2 SC viability, redox response, and phenotypic modulation on PDAM hydrogels

**Fig. 3:**
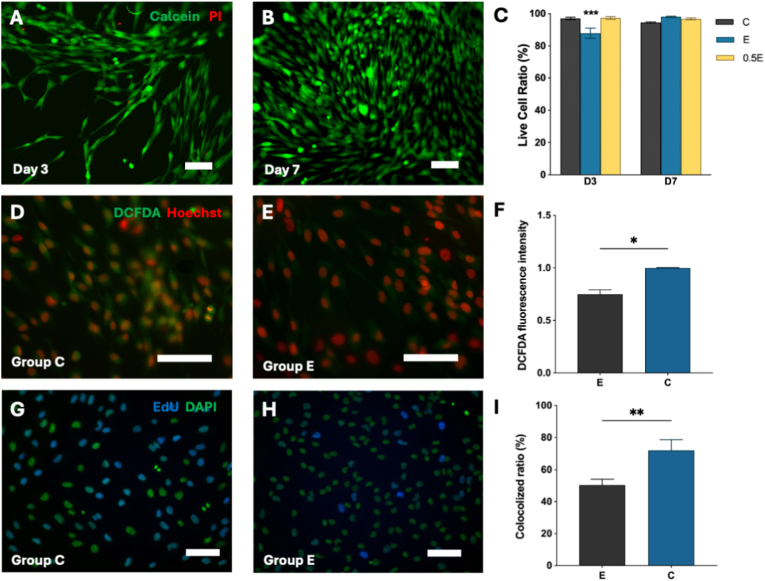
(A-B) Live (green)/dead (red) staining of hSCs on Group E hydrogels at day 3 (A) and day 7 (B). (C) Quantification of live-cell ratio among Group C, Group 0.5 E, and Group E. (D-E) DCFDA staining under oxidative challenge in Group C (D) and Group E (E). (F) Quantification of DCFDA fluorescence intensity. (G-H) EdU staining of proliferating hSCs on Group C (G) and Group E (H). (I) Quantification of EdU and DAPI colocalization ratio. Data shown as mean ± SEM; ∗P < 0.05, ∗∗P < 0.01, ∗∗∗P < 0.001. Scale bars: 100 μm. Sample sizes: n = 4. (For interpretation of the references to colour in this figure legend, the reader is referred to the Web version of this article.)

Because successful peripheral nerve repair relies on the ability of Schwann cells (SCs) to survive oxidative stress and transition between repair and remyelination states, we next examined how the degradable PDAM hydrogels influence SC [27]. All groups supported high cell viability at day 3 and day 7 (Fig. 3A–C, Fig. S4). Although Group E exhibited a statistically lower viability compared with Group C at day 3 (87.9% vs. 97.1%, P = 0.0005), viability remained well above conventional cytocompatibility thresholds and recovered to levels comparable to Group C by day 7, indicating that the initial difference did not compromise long-term cell survival. When exposed to oxidative challenge, hSCs cultured on PDAM hydrogels (Group E) exhibited markedly reduced intracellular 2′,7′-Dichlorodihydrofluorescein diacetate (DCFDA) fluorescence compared with PEGDA controls (Fig. 3D–F), confirming that dopamine release effectively suppressed ROS accumulation. Meanwhile, 5′-ethynyl-2′-deoxyuridine (EdU) incorporation assays revealed a moderate reduction in proliferative activity in Group E (Fig. 3G–H), indicating slower cell-cycle progression under antioxidant conditions. The modest reduction in proliferative activity is consistent with the well-established inverse coupling between SC proliferation and myelination: during normal development, SCs exit the cell cycle before initiating myelin formation, and mTORC1-driven proliferative signaling decreases as myelin gene expression increases [28]. A shift toward lower proliferation on PDAM may therefore reflect progression toward a more differentiated, myelination-competent state rather than a pathological impairment.

**Fig. 4:**
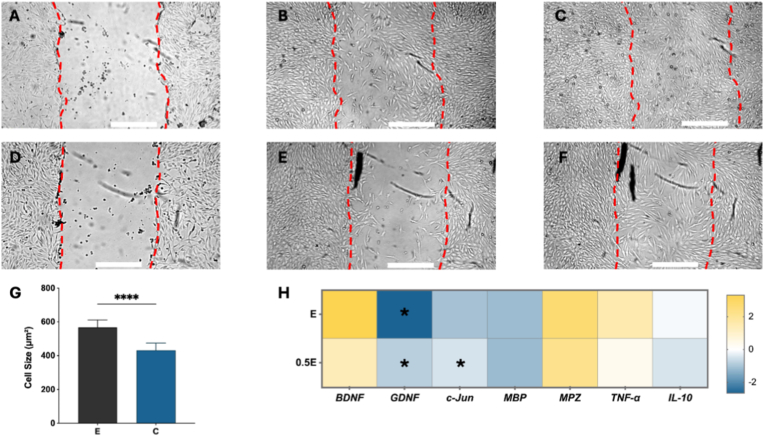
Scratch assay of hSCs incubated with dissolution extract from Group C (A–C) and Group E (D–F) hydrogels at 0, 24, and 48 h respectively. Dashed red lines indicate wound edges. (G) Quantification of cell size. Data shown as mean ± SEM; ∗∗∗∗P < 0.0001. Scale bars: 500 μm. Sample sizes: n = 4. (H) Relative expression of regeneration-associated genes (*BDNF, GDNF, IL-10, Jun, MBP, MPZ, TNF*). Sample sizes: n = 4. Asterisks denote statistically significant differences compared with Group C (one-sample *t*-test, ∗P < 0.05). (For interpretation of the references to colour in this figure legend, the reader is referred to the Web version of this article.)

Scratch migration assays using hydrogel dissolution extracts showed a transient delay in wound closure at 24 h, which recovered by 48 h (Fig. 4A–F and Fig. S2). Cells in Group E displayed enlarged and elongated morphologies (Fig. 4G), consistent with a more mature, repair-associated phenotype [29]. Gene-expression analysis (Fig. 4H) further characterized these shifts in cellular state. Group E hydrogels significantly upregulated gene expression of *BDNF* and *myelin protein zero (MPZ)*, markers associated with neurotrophic signaling and remyelination, respectively. In contrast, expression of *glial-derived neurotrophic factor (GDNF)* and *c-Jun*, markers linked to the early injury response and axonal sprouting were downregulated, while *tumor necrosis factor α (TNF-α)* exhibited a mild but consistent increase, suggesting controlled inflammatory signaling. Expression of *IL-10 (anti-inflammatory cytokine)* decreased modestly, and *myelin basic protein (MBP)* remained relatively stable across conditions.

The PDAM hydrogel supported high cytocompatibility of hSCs and provided intracellular ROS scavenging. The ability of redox-active moieties to buffer oxidative stress and protect hSCs aligns with the recognized importance of ROS balance in maintaining peripheral nerve homeostasis and regeneration [10,30]. EdU incorporation indicated only a mild reduction in proliferation relative to control gels, while scratch assays showed preserved SC migration over 48 h, suggesting that PDAM does not compromise the migratory capacity of hSCs, a property essential for the formation of Büngner bands that guide axonal regrowth in vivo [31,32]. In hSCs, PDAM culture supported migration while shifting cells toward a more mature repair-associated morphology, with enlarged, elongated, and polarized shapes that resemble the elongated “repair” SCs that form longitudinal regeneration tracks in vivo [29,31,32]. At the transcriptional level, PDAM promoted a selectively pro-regenerative program: *BDNF* and *MPZ* were upregulated, *MBP* remained relatively stable, whereas *GDNF* and *c-Jun*, canonical markers of the early injury response and sprouting phase, were reduced, together with a mild shift in inflammatory tone (modest *TNF-α* increase and *IL-10* decrease). This pattern is consistent with a transcriptional shift away from the acute repair state toward markers associated with remyelination competence, while retaining neurotrophic support [29,32]. In the context of diabetic and other metabolically compromised neuropathies, where SC dysfunction, oxidative stress, and loss of trophic support are major drivers of axonal degeneration and pain [10,33], a matrix that sustains BDNF and myelin-gene expression while avoiding chronic “injury-locked” signaling is particularly desirable. Mechanistically, our observation of reduced c-Jun is notable, as c-Jun is the master transcriptional regulator of the repair SC program: high c-Jun levels are required to initiate Wallerian repair but must subsequently decline to permit stable remyelination [34,35]. Recent work further shows that SCs with a persistently activated, mesenchymal-like repair phenotype secrete sFRP1, which engages macrophage HSP90 to drive sustained neuroinflammation and ongoing nerve degeneration after trauma [36]. Thus, the combination of lower *c-Jun* with higher *MPZ* and sustained *BDNF* on PDAM suggests that this hydrogel may favor transcriptional conditions associated with remyelination competence and sustained neurotrophic support, while the reduced c-Jun expression may, in principle, lower the risk of chronically activating sFRP1-linked inflammatory signaling [34,36]. We note that the present gene-expression data, while suggestive, are insufficient to confirm a functional phenotypic transition. Protein-level validation of key markers such as *MPZ* and *MBP*, ultrastructural evidence of myelin wrapping (e.g., transmission electron microscopy), and functional myelination assays using neuron–Schwann cell co-cultures would be required to establish whether the transcriptional shifts observed here translate into actual remyelination competence. Furthermore, while the coordinated downregulation of *GDNF* and *c-Jun* is consistent with attenuation of the acute repair program, we note that GDNF also serves as a potent neurotrophic factor for axonal survival and regeneration independently of its role as a repair SC marker [37]. The sustained upregulation of BDNF on PDAM hydrogels may partially compensate for reduced GDNF-mediated neurotrophic support; however, whether the net balance of these transcriptional changes is favorable for axonal regeneration would need to be evaluated in neuron–Schwann cell co-culture systems or in vivo injury models.

### 2.3 HUVEC viability and angiogenic signaling on PDAM hydrogels

**Fig. 5:**
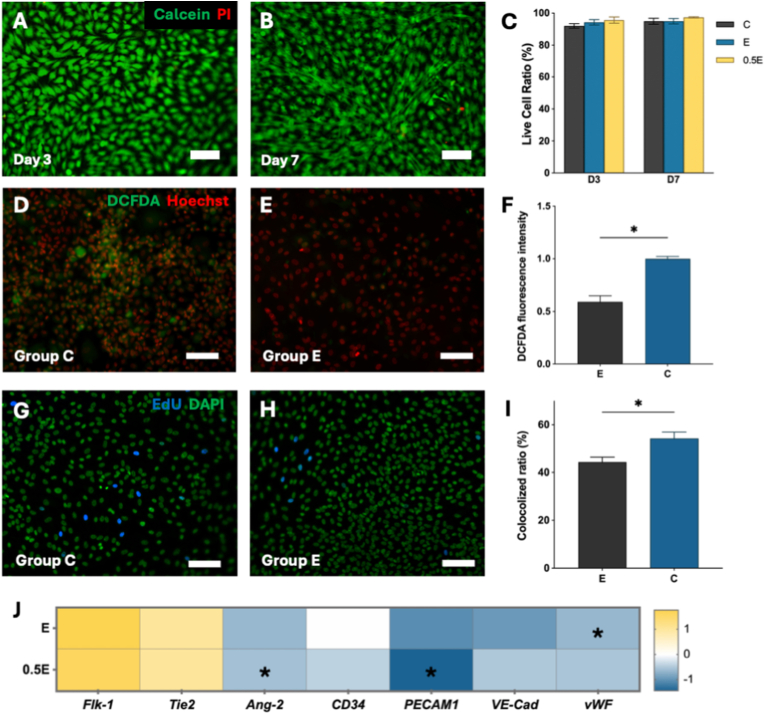
(A–B) Live/dead staining of HUVECs on Group E hydrogels at day 3 (A) and day 7 (B). (C) Quantification of live-cell ratio among Group C, Group 0.5 E, and Group E. (D–E) DCFDA staining under 200 μM H_2_O_2_ in Group C (D) and Group E (E). (F) Quantification of DCFDA fluorescence intensity. (G–H) EdU staining of proliferating HUVECs on Group C (G) and Group E hydrogels (H). (I) Quantification of EdU and DAPI colocalization ratio. Data shown as mean ± SEM; ∗P < 0.05. Scale bars: 100 μm. Sample sizes: n = 4. (J) Relative expression of angiogenic and vessel-stabilization markers (*Flk-1, Tie2, Ang-2, PECAM1, VE-Cad, vWF*). Sample sizes: n = 4. Asterisks denote statistically significant differences compared with Group C (one-sample *t*-test, ∗P < 0.05).

Endothelialization following tissue injury is critical not only for nutrient delivery but also for providing physical guidance scaffolds that direct cellular migration and tissue regeneration, with disruption of organized vascular architecture resulting in failed repair [13,33,38]. To evaluate the endothelial response on PDAM hydrogel formulations, HUVECs were cultured on Group C, Group 0.5 E, and Group E hydrogel. Live/dead staining demonstrated excellent cytocompatibility across all groups, with >90% cell survival maintained through day 7 (Fig. 5A–C, Fig. S4**)**. Under oxidative challenge, HUVECs on Group E hydrogels exhibited markedly reduced DCFDA fluorescence compared with Group C controls (Fig. 5D–F), indicating effective suppression of intracellular ROS by dopamine released during degradation. Similar to what was observed in hSCs, EdU staining revealed a lower proportion of proliferating HUVECs on Group E hydrogels relative to Group C (Fig. 5G–I), suggesting that the antioxidant environment favors cellular stabilization rather than rapid expansion.

Gene-expression analysis further elucidated endothelial signaling changes (Fig. 5J). Quantitative PCR showed upregulated gene expression of *Flk-1* (VEGF receptor 2) and *Tie2*, two key receptors mediating angiogenic sprouting and vessel maturation, accompanied by modest downregulation of *Ang-2*, *PECAM1*, *VE-Cad*, and *vWF*, which are associated with vascular barrier integrity [39]. These findings indicate that PDAM hydrogels support endothelial survival and oxidative protection while transiently promoting a pro-angiogenic transcriptional profile.

**Fig. 6:**
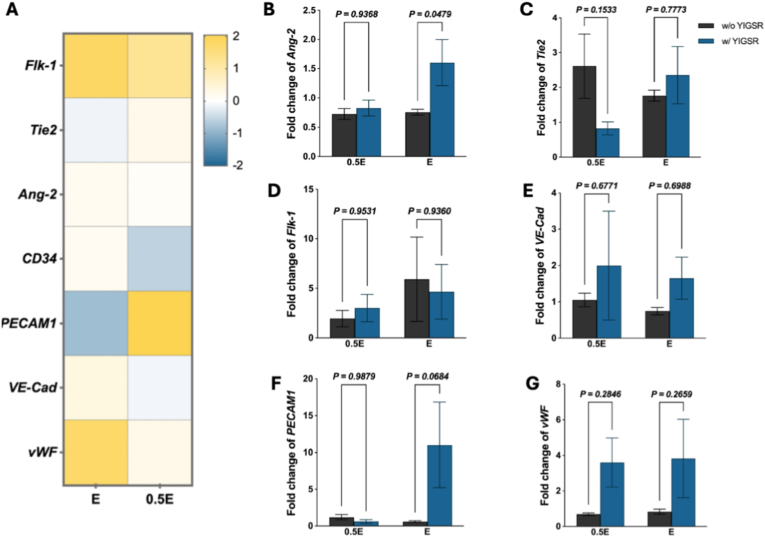
(A) Relative expression of angiogenic and vessel-stabilization markers (*Flk-1, Tie2, Ang-2, PECAM1, VE-Cad, vWF*) following CYIGSR functionalization of hydrogels. (B-G) Relative fold-change in expression of *Flk-1, Tie2, Ang-2, PECAM1, VE-Cad,* and *vWF* for CYIGSR-modified versus unmodified PDAM hydrogels. Data shown as mean ± SEM. Sample sizes: n = 4.

To enhance vascular stabilization cues, hydrogels were subsequently functionalized with the laminin-derived peptide CYIGSR, a sequence known to promote endothelial adhesion and basement-membrane organization [40]. Fluorescent imaging confirmed successful conjugation of CYIGSR to the hydrogel surface (Fig. S5). As shown in Fig. 6A, gene expression analysis revealed that CYIGSR functionalization upregulated both *Flk-1* and *Tie2*, together with increases in *Ang-2, PECAM1, VE-Cad*, and *vWF*, restoring expression balance between angiogenic activation and vessel-stabilization pathways (Fig. 6B–G).

In HUVECs, except for good cytocompatibility and ROS scavenging capability, PDAM hydrogels also modulated endothelial phenotype in a manner consistent with transcriptional programs associated with angiogenic activation and vessel maturation. Without peptide functionalization, PDAM increased expression of *Flk-1* and *Tie2*, key signaling nodes for VEGF/PlGF-driven sprouting and Ang–Tie–mediated remodeling, while modestly reducing *Ang-2*, *PECAM1*, *VE-Cadherin*, and *vWF*, markers associated with endothelial junctions, barrier integrity, and luminal organization [39]. This transcriptional pattern suggests that antioxidant capacity alone is insufficient to establish functional vessels: PDAM alone biases endothelial cells toward a transcriptional profile associated with an activated, sprouting state, with reduced expression of junction and barrier markers suggesting that antioxidant signaling alone may be insufficient to support full vascular stabilization at the gene-expression level. Upon CYIGSR functionalization, we observed a coordinated rise in both *Flk-1* and *Tie2* together with restoration or enhancement of *Ang-2*, *PECAM1*, *VE-Cadherin*, and *vWF* expression, in line with the established role of laminin-derived motifs in promoting endothelial adhesion, basement-membrane assembly, and vessel stabilization [40]. This suggests that bioactive signals (peptides) combined with the antioxidant scaffold may be necessary to achieve balanced expression of both angiogenic and vessel-stabilization markers, a prerequisite for constructing a functional neurovascular unit in vivo, effectively highlighting the value of our “flexible design” strategy wherein material properties and biochemical cues can be independently tuned to guide coordinated tissue remodeling. Given that regenerating axons and repair Schwann cells use blood vessels as structural guides and depend on adequate perfusion, and that VEGF/PlGF–VEGFR and Ang–Tie2 pathways are central to coordinated neurovascular remodeling after nerve injury, the ability of PDAM–CYIGSR constructs to modulate endothelial gene expression toward both angiogenic and stabilization programs provides a potentially complementary axis of control that warrants further in vivo investigation in complex, neuropathic environments [35].

### 2.4 Geometric and therapeutic customizability of PDAM hydrogels

**Fig. 7:**
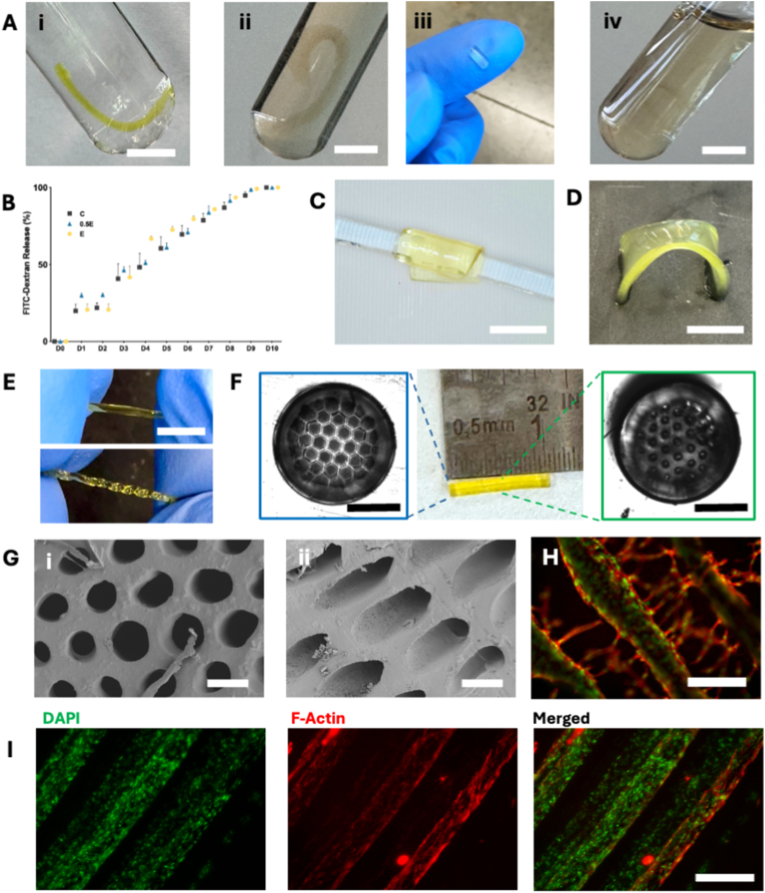
(A) Tubular constructs of 2 cm immediately after printing (i), after 1 month (ii), after 1 month with a segment excised to demonstrate maintained structural integrity (iii), and after 2 months (iv). Scale bars: 1 cm. (B) FITC-dextran release profile. (C) PDAM sheet (200 μm thick) wrapped around a 3D-printed phantom (1 mm diameter). Scale bars: 500 μm. (D) Digital image of conduit maintaining structural integrity under 180-degree bending deformation. Scale bars: 5 mm. (E) Reversible torsional testing showing the conduit tolerating 7-8 twists without fracture (i, ii). Scale bars: 1 cm. (F) Conduit with hexagonal microchannels: overall length, top view (left), and cross-sectional view after bisecting (right). Scale bars: 500 μm. (G) SEM images showing detailed microchannel architecture and smooth internal walls. Scale bars: 200 μm. (H) Merged fluorescence image of hSCs aligned along printed grooves, with DAPI (green) and F-actin (red) demonstrating topography-guided cell alignment. (I) F-actin staining within microchannel conduits showing DAPI (green), F-actin (red), and merged images. Scale bars: 200 μm. (For interpretation of the references to colour in this figure legend, the reader is referred to the Web version of this article.)

To demonstrate the versatility of the PDAM hydrogel platform, we evaluated its capability to support diverse geometries and tunable therapeutic cargo release relevant to peripheral nerve repair. Straight tubular constructs approximately 2 cm in length were fabricated by DLP printing to represent long-gap nerve conduits and incubated in PBS at 37 °C to assess degradation under physiologically relevant conditions (Fig. 7A). As-fabricated tubes (Fig. 7A–i) maintained their structural integrity immediately after printing. After 1 month (Fig. 7A–ii), tubes showed early-stage degradation while retaining their overall geometry. When a segment was excised at the 1-month time point (Fig. 7A–iii), the material demonstrated preserved structural integrity, confirming the conduit's ability to maintain mechanical support during the critical early phase of nerve regeneration. By 2 months (Fig. 7A–iv), the tubes had substantially degraded and lost their defined shape in the liquid environment. This extended timeline is consistent with the gravimetric data in Fig. 1A, where degradation was still ongoing at the 18-day endpoint for smaller specimens; the slower resorption of printed conduits reflects their greater bulk volume and lower surface-area-to-volume ratio. Together, these results demonstrate the material's capacity for controlled resorption over timescales relevant to peripheral nerve regeneration. To test the potential of this material as a drug delivery vehicle, the hydrogels were loaded with FITC-dextran (40 kDa) as a model macromolecular therapeutic to mimic growth factor as reported in the literature [41,42]. FITC-dextran was released steadily during the first 7 days with no obvious burst release (Fig. 7B). All groups exhibited gradual release reaching complete elution by day 10, with a near-linear cumulative release profile of approximately 10% per day during the first week. There was no significant difference between the three groups, though Group E showed a trend toward faster degradation.

To prove the high customizability of this material for nerve repair, we first demonstrated its potential as a nerve wrap (Fig. 7C). We printed a 200 μm-thick sheet and wrapped it around a phantom with a diameter of 1 mm. The wrap adhered uniformly to the surface without visible delamination or tearing and conformed closely to the phantom curvature. As shown in Movies S1 and S2, the wraps possessed intrinsic surface adhesion sufficient to resist detachment when lifted or repositioned with tweezers, maintaining their folded configuration throughout handling.

Mechanical flexibility was further examined through torsional and bending deformation tests. Planar slabs maintained integrity when bent 180° without mid-section fracture (Fig. 7D), while printed tubes tolerated seven consecutive twisting cycles without visible cracking or structural failure (Fig. 7E), demonstrating the material's resilience under physiological stresses that peripheral nerve conduits may experience during implantation and patient movement.

To further demonstrate geometric fidelity and design flexibility, we printed a 1 cm conduit containing internal hexagonal microchannels (around 120 μm in diameter), as shown in Fig. 7F. The microchannel diameter of approximately 120 μm was selected to approximate the dimensions of endoneurial tubes and small fascicles in peripheral nerves (typically 50–200 μm), providing sufficient space for SC infiltration and alignment while remaining within the resolution capabilities of DLP printing [4]. Channel integrity was verified by examining the ends of the conduit as well as cross-sections in the middle using Leica brightfield microscopy (Fig. 7F) and SEM (Fig. 7G), confirming that the PEGDA-Do bioink can generate microscale architectures that could be conducive to guiding cell alignment. Using hSCs as a representative model, we seeded cells on printed grooves and observed cell alignment along the groove geometry (Fig. 7H). The merged fluorescence image shows coordinated cellular organization with nuclei and F-actin aligned along the topographical features. Such cell alignment was also observed within conduits containing microchannels, as shown in the cytoskeleton staining (Fig. 7I). DAPI (nucleus, green) and F-actin (red) staining revealed that hSCs proliferated and expanded along channels with diameters of approximately 100 μm. The merged image demonstrates elongated cell morphology and alignment parallel to the channel orientation, highlighting the potential of PDAM constructs to direct hSCs behavior for enhanced nerve regeneration within three-dimensional microenvironments.

## 3 Discussion

Peripheral nerve repair remains hindered by the lack of biomaterials that can couple tunable degradation with biochemical functionality and structural adaptability. Conductive hydrogels (e.g., polypyrrole- or graphene-based composites) provide electrical cues to promote SC responses but typically lack programmable degradation kinetics and do not address oxidative stress. Peptide-functionalized matrices offer targeted biochemical signaling but often rely on non-degradable or poorly tunable backbones. Aligned electrospun fiber scaffolds provide excellent topographical guidance for SC alignment but are difficult to customize into patient-specific geometries. Multichannel conduits achieve structural complexity but frequently lack bioactive functionality beyond physical guidance. More broadly, most current platforms focus on axon guidance or late-stage trophic cues while offering only coarse control over degradation kinetics and the hostile post-injury niche, leading to inconsistent outcomes in severe or metabolically compromised injuries [43].

Herein, we developed a degradable PDAM hydrogel with tunable degradation and dopamine release, designed to address these limitations through reversible aza-Michael chemistry and composition-dependent crosslinking. By varying the proportion of PEGDA-Do within the bioink, we were able to modulate the overall degradation behavior and the amount of dopamine release, providing a controllable redox environment that can adapt to different therapeutic timeframes. This tunability is particularly advantageous for aligning scaffold degradation with the regenerative rate of target tissues and offers flexibility in designing nerve repair strategies across varying injury models.

Our results demonstrated that PDAM hydrogels maintained mechanical characteristics comparable to native peripheral nerve tissues while exhibiting exceptional flexibility and resilience. These properties enabled the formation of stable tubular or wrap configurations that withstood multiple twisting and bending cycles, supporting their potential utility during implantation and long-term dynamic movement. Biologically, the PDAM hydrogel supported high cytocompatibility of both hSCs and HUVECs and provided intracellular ROS scavenging, consistent with the antioxidant function of dopamine. In hSCs, PDAM upregulated BDNF and MPZ with reduced c-Jun and GDNF, a transcriptional pattern consistent with progression away from the “injury-locked” state toward remyelination-associated gene expression and a transition particularly critical in diabetic neuropathy where persistently high c-Jun prevents stable myelin repair. In HUVECs, PDAM alone increased Flk-1 and Tie2 expression while modestly reducing junction and barrier markers, biasing cells toward an activated, sprouting state. CYIGSR functionalization coordinated upregulation of both sprouting receptors and stabilization markers (Ang-2, PECAM1, VE-Cadherin, vWF), promoting vessel maturation. Together, PDAM hydrogels modulate gene expression in both hSCs and HUVECs in patterns suggestive of coordinated pro-regenerative and pro-angiogenic transcriptional programs, providing an in vitro foundation that warrants future in vivo validation of functional neurovascular remodeling and remyelination outcomes.

The hydrogel's compatibility with DLP printing further expands its translational potential. The ability to fabricate conduits and microchannel architectures with high precision allows for integration of physical guidance cues and local drug reservoirs within a single construct. The system also demonstrated sustained FITC-dextran release, confirming its suitability for localized delivery of therapeutic agents such as neurotrophic proteins or anti-inflammatory compounds. Moreover, its printable, adhesive, and flexible nature enables direct adaptation as therapeutic wraps, which, when combined with its hSCs–priming capability, may serve as a modular platform for treating peripheral neuropathy, including diabetic neuropathy, where prolonged oxidative and inflammatory stress impede regeneration and where SCs remyelination capability is insufficient [10].

## 4 Conclusion

In summary, the PDAM hydrogel developed in this study provides a customizable and multifunctional platform with in vitro properties supportive of peripheral nerve repair applications. Its composition-tuned degradation and dopamine release offer programmable control over scaffold lifespan and redox modulation; its mechanical compliance and biocompatibility ensure functional integration; and its printability enables geometrical versatility for both conduit and wrap-based applications. Together, these findings establish a promising foundation for developing adaptive nerve repair materials tailored to both acute injury and chronic neuropathic conditions. We acknowledge that the present study is limited to in vitro characterization of Schwann cell and endothelial cell responses and does not include neuronal functional assays such as neurite outgrowth or dorsal root ganglion co-culture experiments. While the hSC and HUVEC data establish that PDAM supports the cellular components of the pre-axonal regenerative niche, functional validation of axonal extension, neuron–Schwann cell interactions, and nerve regeneration outcomes will require future in vivo studies in peripheral nerve injury models.

## 5 Experimental section

### 5.1 Chemicals

PEGDA (Mn 575 Da and 700 Da), acrylamide, dopamine hydrochloride, hydroquinone, tartrazine (TART), dimethylformamide (DMF), triethylamine (TEA), ethyl acetate, n-hexane, and vitamin E (α-tocopherol) were obtained from Sigma-Aldrich (St. Louis, MO, USA). Lithium phenyl-2,4,6-trimethylbenzoylphosphinate (LAP) was purchased from Tokyo Chemical Industry (Tokyo, Japan). DCFDA was purchased from MedChemExpress. Dulbecco's phosphate-buffered saline (DPBS) and trypsin-EDTA were from Gibco (Thermo Fisher Scientific).

### 5.2 Bioink composition

GelMA was synthesized following published protocol [44,45]. PEGDA-Do was synthesized via aza-Michael addition between dopamine hydrochloride and PEGDA (Mn 575 Da) following Liang et al. with modification [19]. Briefly, hydroquinone (25 mg, 0.227 mmol) and dopamine·HCl (25 g, 0.132 mol) were charged into a 500 mL Schlenk flask, purged with nitrogen, and dissolved in anhydrous DMF (100 mL). PEGDA (Mn 575 Da, 76 g, 0.132 mol) was added dropwise under nitrogen, followed by TEA (13.5 g, 0.133 mol). The mixture was heated to 85 °C with stirring for 24 h. To maximize residual acrylate functionality, additional PEGDA (Mn 575 Da, 15 g, 0.026 mol) was introduced under inert atmosphere and the reaction was continued for 48 h. The mixture was cooled to 4 °C for 30 min, filtered, and the yellow filtrate was precipitated five times into ethyl acetate:n-hexane (1:1 v/v; total 600 mL). The precipitate was collected by centrifugation, supplemented with vitamin E (12 mg) as a stabilizer, and vacuum-dried to afford PEGDA-Do as a pale-yellow liquid. Gel permeation chromatography (Shimadsu™ LC-2050 GPC) was used to analyze the polymer molecular weight as calibrated by poly (methyl methacrylate) standards. ^1^H NMR was performed using a JEOL-500 NMR with Delta NMR processing software with the chemical shifts referenced to chloroform (CDCl3) [19,46] (Fig. S1).

A base resin was prepared in DPBS containing acrylamide (13% w/v), LAP (0.6% w/v) as photoinitiator, TART (0.075% w/v) as photoabsorber, and GelMA (1% w/v) to enhance cell adhesion. Crosslinker content was controlled at a monomer: crosslinker molar ratio of 100:0.8. Three formulations were used: (1) Group C. PEGDA as crosslinker. (2) Group E. PEGDA-Do as crosslinker. (3) Group 0.5 E. PEGDA and PEGDA-Do as crosslinkers with a mix ratio of 1:1 (molar), same total crosslinker content.

### 5.3 DLP-based 3D printing

Bioprinted constructs were fabricated on an in-house micro-continuous optical printing (μCOP) DLP system as previously described [46,47]. For planar slabs and 2 mm cubes used for cell seeding, printing was performed in a fixed-gap mode: polydimethylsiloxane (PDMS) spacers (250 μm) and a PDMS-coated coverslip defined the resin thickness and surface finish, the z-stage remained stationary, and a digital micromirror device (DMD) projected the UV pattern to cure the bioink uniformly across the focal plane. In contrast, tubular geometries were produced in a bottom-up configuration using continuous μCOP: the DMD projected the layer pattern into a PDMS-coated reservoir while the z-axis actuator translated upward at 0.01 mm/s, enabling progressive photopolymerization at the optical focal plane and seamless interlayer adhesion throughout the build.

### 5.4 Degradation test

To test the degradation of different compositions, several samples of equal mass obtained by DLP printing were placed in tubes containing 5 mL Milli-Q water and incubated at 37 °C. At each sampling time point, the samples were removed from the incubation medium and vacuum dried overnight and weighed, meanwhile, 200 μL of supernatant was collected for absorbance measurements at 280 nm using a Tecan Infinite 200 PRO. For the long-term degradation assessment of printed tubular conduits, constructs were incubated in 5 mL PBS at 37 °C and monitored for 2 months for structural integrity.

### 5.5 Scanning electron microscopy (SEM)

Specimens were lyophilized prior to SEM imaging. Dried samples were mounted on aluminum stubs using carbon adhesive tape and sputter-coated with iridium using an Emitech K575X sputter coater to ensure electrical conductivity. High-resolution imaging was performed using a Zeiss Sigma 500 field emission scanning electron microscope operated at 3 kV.

### 5.6 Mechanical property testing

Young's modulus was measured on a MicroSquisher (CellScale) in displacement-controlled ramp mode following the manufacturer's protocol. DLP-printed cylindrical specimens (0.5 mm diameter × 0.5 mm height) were compressed to a 100 μm displacement with 50 s loading and 50 s recovery phases, corresponding to a displacement rate of 2 μm/s and an engineering strain rate of 0.004/s (20% strain over 50 s). Uniaxial tensile properties were obtained on an Instron 5965 (10 N load cell; Norwood, MA). Dog-bone specimens were printed for formulation comparisons and tested at a constant strain rate of 0.5% per second until failure.

Equilibrium swelling was evaluated on DLP-printed 250 μm planar slabs. Acellular constructs were dehydrated overnight at 37 °C and imaged, then rehydrated in DPBS and re-imaged at predetermined time points over 8 days on a Leica DMI 6000-B microscope. Swelling ratios were calculated as A_wet_/A_dry_ using ImageJ [48].

### 5.7 Cell culture

HUVECs were obtained from CellApplication Inc. and cultured according to manufacturer specifications. Cells were maintained in endothelial growth medium (211-500, Cell Application Inc.) with medium changes every 2 days and subcultured at a 1:5 ratio every 4 days. Only cells below passage 7 were used to ensure phenotypic stability. For seeding procedures, confluent HUVECs were washed with DPBS and detached using 0.25 trypsin-EDTA solution for 3 min at 37 °C in a humidified CO_2_ incubator. Enzymatic activity was neutralized with complete culture medium, and cells were resuspended to a final seeding density of 1.5 × 10^6^ cells/mL.

Human Schwann cells (hSCs) were obtained from ATCC. Cells were grown in DMEM containing 10% FBS, 4 mM glutamine, and 100 U/mL and 100 μg/mL penicillin-streptomycin (complete DMEM). Only cells below passage 10 were used to ensure phenotypic stability. For seeding procedures, confluent hSCs were washed with DPBS and detached using 0.25% trypsin-EDTA solution for 3 min at 37 °C in a humidified CO_2_ incubator. Enzymatic activity was neutralized with complete culture medium, and cells were resuspended to a final seeding density of 1 × 10^5^ cells/mL.

### 5.8 Viability assay

Cell viability was evaluated using Live/Dead fluorescent staining at predetermined time points. Constructs were washed twice with DPBS and incubated with 2 μM calcein AM (C3099, Invitrogen) and 3 μM propidium iodide (P3566, Invitrogen) in fresh culture medium for 30 min under standard culture conditions (37 °C, 5% CO_2_). Following incubation, samples were washed twice with DPBS and immediately imaged using a Leica DMI6000B fluorescence microscope. Live cell ratios were quantified from fluorescence images using ImageJ software. Live cells (calcein-positive) and dead cells (propidium iodide-positive) were counted, and viability was calculated as (number of live cells/total number of cells) × 100%. Four randomly captured images per condition were analyzed for quantification [44,49,50].

### 5.9 Antioxidant capacity evaluation assay

To evaluate the antioxidant capacity of hydrogel degradation products, DLP-printed hydrogel slabs of identical dimensions from Group C, Group 0.5 E, and Group E were immersed in culture medium for 24 h to generate hydrogel-incubated medium. HUVECs were seeded on standard tissue culture plates, cultured for 24 h, and then treated with the corresponding hydrogel-incubated medium supplemented with 200 μM H_2_O_2_ for 24 h before co-staining with DCFDA and Hoechst as described below. To further evaluate the antioxidant capacity of the PDAM hydrogel under a highly oxidative microenvironment, cells were seeded on the hydrogel surface and cultured for 24 h. The culture medium was then replaced with fresh medium containing 200 μM H_2_O_2_ to induce oxidative stress [51,52]. After an additional 24 h of incubation, cells from each group were co-stained with the DCFDA probe (HY-D0940, MCE) to detect intracellular ROS and with Hoechst for nuclear visualization. Fluorescence images were captured using a fluorescence microscope and DCFDA fluorescence intensity was quantified using ImageJ software. The mean fluorescence intensity of Group C was normalized to 1, and the intensities of Group 0.5 E and Group E were expressed as ratios relative to Group C.

### 5.10 Immunofluorescence assay

EdU staining was performed using the Click-iT™ EdU Imaging Kit (C10640, Invitrogen) according to the manufacturer's instructions. Cells were seeded and cultured for 24 h, EdU was added to the culture medium at a final concentration of 10 μM. After an additional 2 h culture for EdU incorporation, the medium was then removed and samples were fixed with 4% paraformaldehyde for 15 min at room temperature. Following fixation, the samples were rinsed twice with 2% BSA for 5 min each and permeabilized with 0.5% Triton X-100 for 20 min. After two additional rinses with 2% BSA, the samples were incubated with the Click-iT reaction cocktail (reaction buffer, CuSO_4_, Alexa Fluor azide, and reaction buffer additive) for 30 min at room temperature. Finally, the samples were rinsed once with 2% BSA for 5 min before imaging.

### 5.11 Cell scratch assay

After hSCs were cultured as a monolayer on 24-well plates, a 200 μL sterile pipette tip was used to create a linear wound at the midline. Subsequently, the remaining cell debris was removed using PBS, and cells were incubated with dissolution extract of PDAM hydrogel for 3 days; micrographs of the cells were recorded daily. Wound closure was quantified using ImageJ software following the MRI Wound Healing Tool method (Montpellier RIO Imaging). Cell size was measured from brightfield micrographs by manually tracing individual cell boundaries using the freehand selection tool in ImageJ, with at least 30 cells per group analyzed.

### 5.12 Quantitative real-time PCR

RNA extraction was performed using TRIzol reagent (Life Technologies). To obtain sufficient RNA, 12 constructs per group were collected and divided into 4 biological replicates. Briefly, bioprinted constructs were lysed in TRIzol and RNA was purified using the Direct-zol RNA Microprep spin-column kit (Cat. #R2060, Zymo Research). RNA was resuspended in RNase-free water and quantified on a Tecan plate reader. For qRT-PCR, 200 ng of RNA was reverse-transcribed into cDNA using the First Strand cDNA Synthesis Kit (Cat. #E6300S, New England BioLabs). qRT-PCR was then carried out with Luna® Universal qPCR Master Mix (Cat. #M3003S, New England BioLabs) on a QuantStudio 3 system. RNA samples were collected at day 3 after seeding on the hydrogel constructs. Relative gene expression was calculated using the comparative Ct (ΔΔCt) method by normalizing threshold cycle (Ct) values to a housekeeping gene, with Group C as the reference control. Forward and reverse primers were purchased from Integrated DNA Technologies, and sequences are listed in Table S1.

### 5.13 Statistics and reproducibility

Statistical analyses were performed using GraphPad Prism 9 (La Jolla, CA, USA). Data normality was assessed using the Shapiro-Wilk test. Multiple group comparisons were analyzed by one-way ANOVA followed by Sidak's post hoc test. Statistical significance was set at *P* < 0.05.

## CRediT authorship contribution statement

**Lin Huang:** Conceptualization, Data curation, Formal analysis, Investigation, Methodology, Validation, Visualization, Writing – original draft. **Ting-Yu Lu:** Conceptualization, Data curation, Formal analysis, Investigation, Methodology, Validation, Visualization. **Emma Berman:** Data curation, Investigation, Methodology. **Alexander Park:** Conceptualization, Investigation, Methodology. **Katarina Ercegovac:** Data curation, Investigation, Methodology. **Jacob Schimelman:** Resources. **Shaochen Chen:** Conceptualization, Funding acquisition, Project administration, Resources, Supervision, Writing – review & editing.

## Declaration of competing interest

The authors declare the following financial interests/personal relationships which may be considered as potential competing interests: Shaochen Chen reports financial support was provided by National Institutes of Health. Shaochen Chen reports a relationship with National Institutes of Health that includes: funding grants. Shaochen Chen has patent pending to University of California San Diego. If there are other authors, they declare that they have no known competing financial interests or personal relationships that could have appeared to influence the work reported in this paper.

## Data Availability

Data will be made available on request.
