# Supplementary material for: A degradable PEGDA-dopamine hydrogel with ROS scavenging capacity supports flexible design for nerve repair
Source: Mater Today Bio. 2026 May 5;38:103203. doi: 10.1016/j.mtbio.2026.103203 (PMC13156770; doi:10.1016/j.mtbio.2026.103203)
Supplement: Multimedia component 1 [file mmc1.docx]

**Supplementary Information**

**A Degradable PEGDA-Dopamine Hydrogel with ROS Scavenging Capacity Supports Flexible Design for Nerve Repair**

*Lin Huang, Ting-Yu Lu, Emma Berman, Alexander Park, Katarina Ercegovac, Jacob Schimelman, Shaochen Chen^*^*

L. Huang, T. Lu, J. Schimelman and S. Chen

Department of Chemical and Nano Engineering, University of California San Diego, La Jolla, USA

Email: [shc064@ucsd.edu](mailto:shc064@ucsd.edu)

E. Berman

Department of Bioengineering, University of California, Berkeley, Berkeley, USA

A. Park, K. Ercegovac and S. Chen

Department of Bioengineering, University of California San Diego, La Jolla, USA

*Correspondence: Shaochen Chen; email: [shc064@ucsd.edu](mailto:shc064@ucsd.edu)


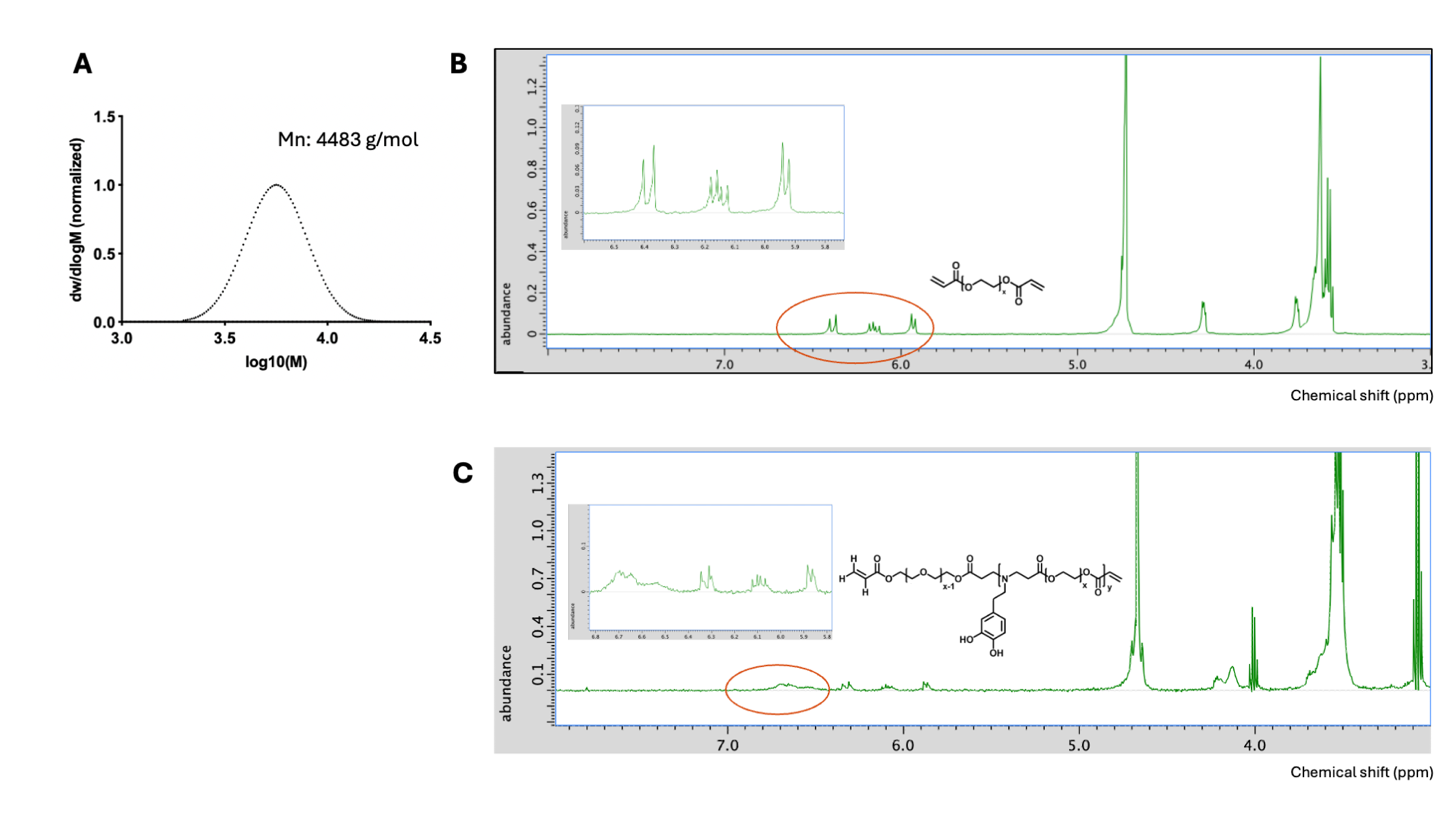


Figure S1. (A) Gel permeation chromatography (GPC) analysis of PEGDA-Do. (B-C) ^1^H NMR spectra of PEGDA (B) and PEGDA-Do (C)


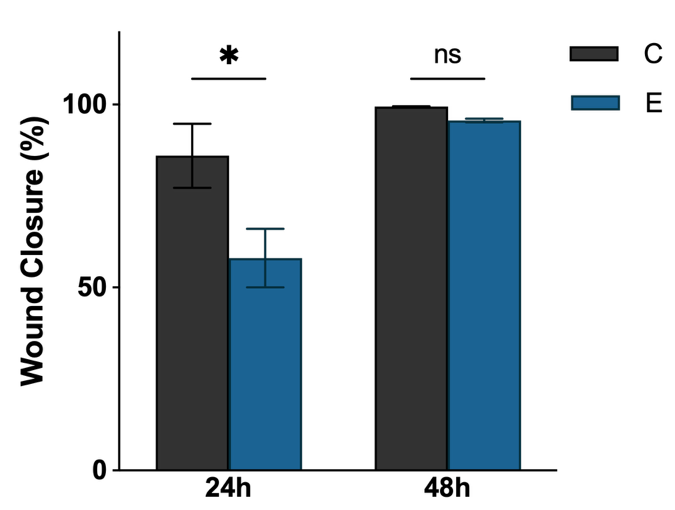


Figure S2. Quantification of wound closure in the scratch migration assay for Group C and Group E at 24 h and 48 h. Data shown as mean ± SEM; *P < 0.05, (n = 3).


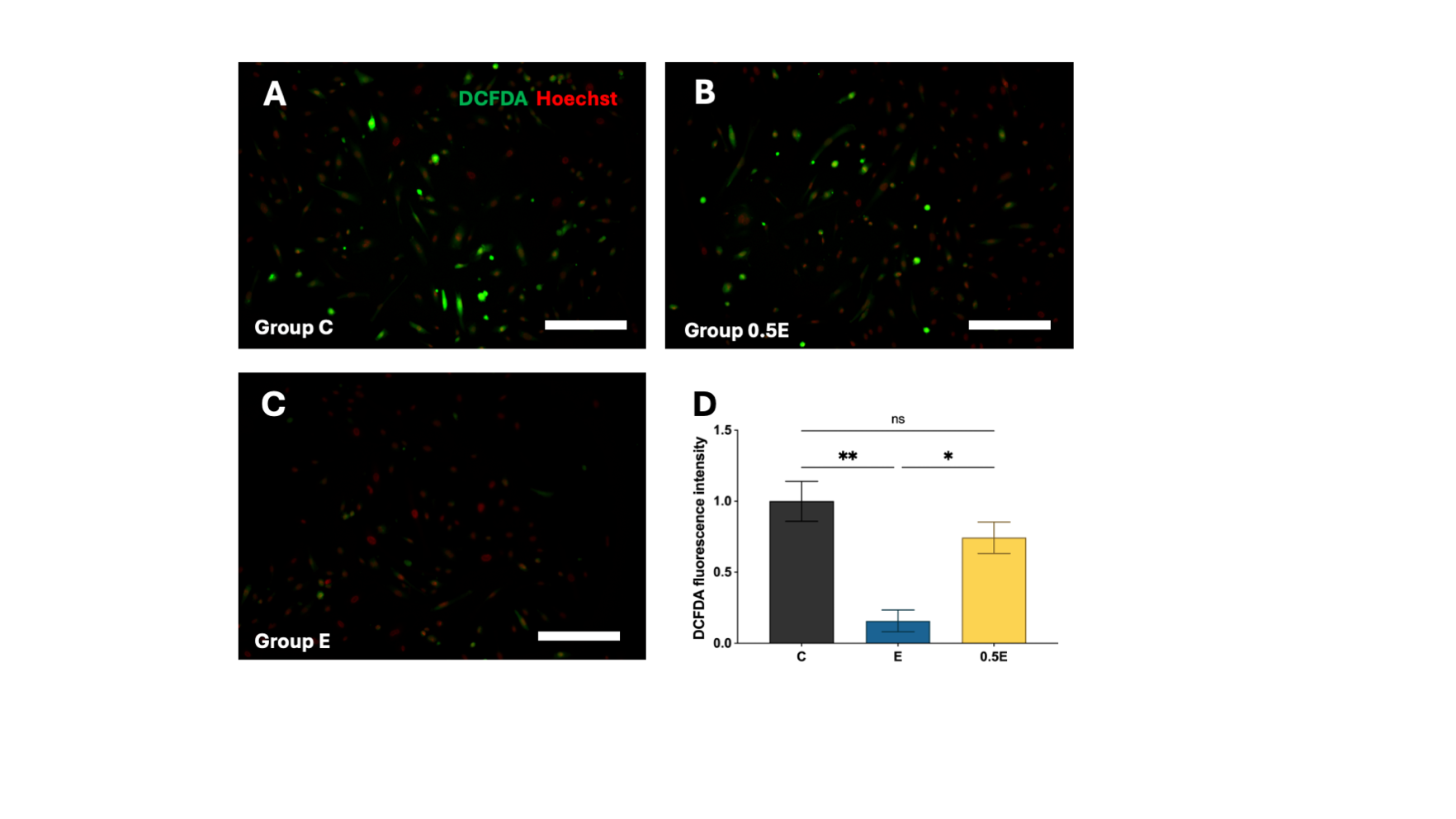


Figure S3. DCFDA (green) and Hoechst (red) staining of HUVECs cultured on tissue culture plates with hydrogel-incubated medium from Group C (A), Group 0.5E (B), and Group E (C) following oxidative challenge with 200 µM H₂O₂. Scale bars: 250 µm. (D) Quantification of DCFDA fluorescence intensity. Sample sizes: n=4.


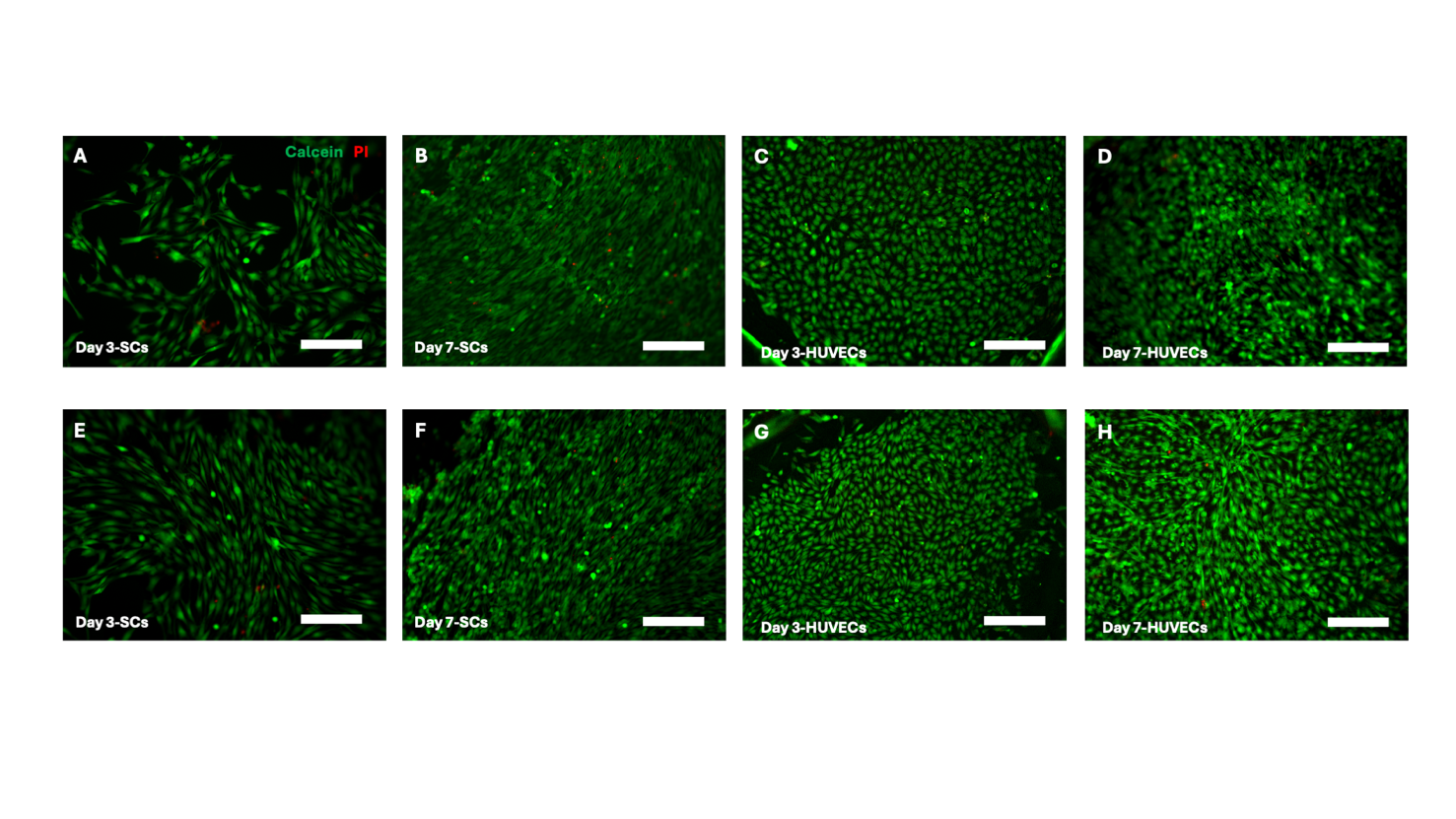


Figure S4. Live/dead staining of hSCs and HUVECs on Group C (A–D) and Group 0.5E (E–H) hydrogels. Scale bars: 250 µm.


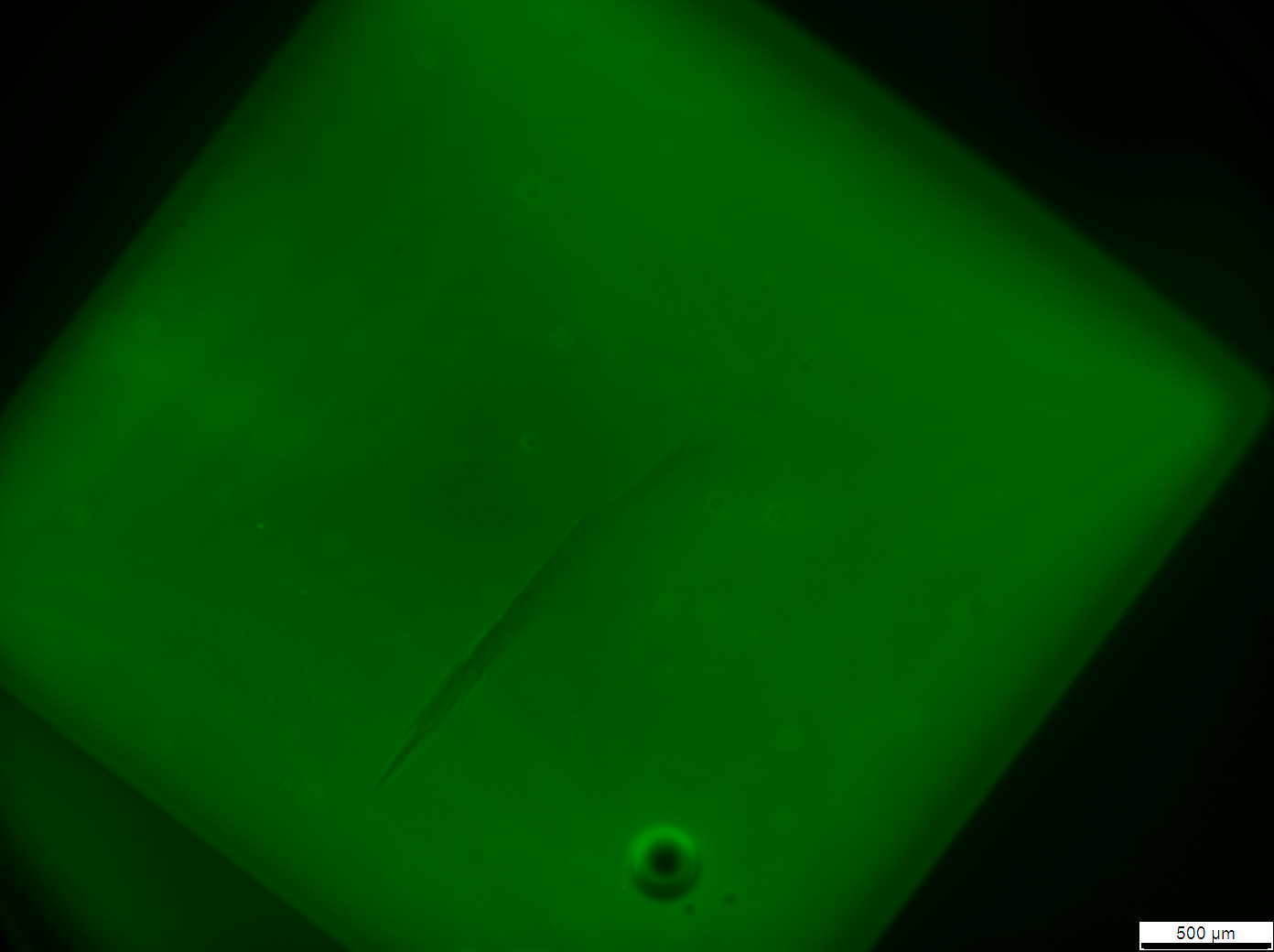


Figure S5 Fluorescent image of FAM-CYIGSR–conjugated hydrogel confirming successful surface modification.

Table S1. qRT-PCR primers

| **Gene** | **Primer sequences (forward and reverse)** |
| --- | --- |
| **MBP** | 5’- AATCGGCTCACAAGGGATTCA-3’  3’- TCCTCCCAGCTTAAAGATTTTGG-5’ |
| **BDNF** | 5’- TCATACTTCGGTTGCATGAAGG-3’  3’- AGACCTCTCGAACCTGCCC-5’ |
| **c-Jun** | 5’- CCTTCTACGACGATGCCCTC-3’  3’- GGTTCAAGGTCATGCTCTGTTT-5’ |
| **GDNF** | 5’- CCAGTGACTCCAATATGCCTG-3’  3’- CTCTGCGACCTTTCCCTCTG-5’ |
| **MPZ** | 5’- CTGCTCCTTCTGGTCCAGTG-3’  3’- GCGCTTCGAGGAGTCCTTAG-5’ |
| **GAPDH** | 5’- CCGCATCTTCTTGTGCAGTG-3’  3’- ACCAGCTTCCCATTCTCAGC-5’ |
| **IL-10** | 5’- AAGGGTTACTTGGGTTGCCA-3’  3’- AGACACCTTTGTCTTGGAGCTTA-5’ |
| **TNF-α** | 5’- GTAGCCCACGTCGTAGCAAA-3’  3’- GGCTGGGTAGAGAACGGATG-5’ |
| **PECAM1** | 5’-GCTGTTGGTGGAAGGAGTGC-3’  3’- GAAGTTGGCTGGAGGTGCTC-5’ |
| **CD34** | 5’-TGAAGCCTAGCCTGTCACCT-3’  3’-CGCACAGCTGGAGGTCTTAT-5’ |
| **VE-Cad** | 5’-ACGGGATGACCAAGTACAGC-3’  3’-ACACACTTTGGGCTGGTAGG-5’ |
| **Flk-1** | 5’-CTGGCATGGTCTTCTGTGAAGCA-3’  3’-AATACCAGTGGATGTGATGGCGG-5’ |
| **vWF** | 5’-ATGTTGTGGGAGATGTTTGC-3’  3’-GCAGATAAGAGCTCAGCCTT-5’ |
| **Tie2** | 5’-CCCATTTGCAAAGCTTCTGGCTGGC-3’  3’-TGTGAAGCGTCTCACAGGTCCAGGATG-5’ |
| **Ang-2** | 5’-GGATCTGGGGAGAGAGGAAC-3’  3’-CTCTGCACCGAGTCATCGTA-5’ |
| **GAPDH** | 5’-AGCCACATCGCTCAGACACC-3’  3’-GTACTCAGCGCCAGCATCG-5’ |
